# Effects of Respiratory Motion on Y-90 PET Dosimetry for SIRT

**DOI:** 10.3390/diagnostics12010194

**Published:** 2022-01-14

**Authors:** Matthew D. Walker, Jonathan I. Gear, Allison J. Craig, Daniel R. McGowan

**Affiliations:** 1 Department of Medical Physics and Clinical Engineering, Churchill Hospital, Oxford University Hospitals NHS Foundation Trust, Oxford OX3 7LE, UK; daniel.mcgowan@oncology.ox.ac.uk; 2 Joint Department of Physics, The Royal Marsden NHS Foundation Trust and Institute of Cancer Research, Sutton SM2 5PT, UK; jonathan.gear@icr.ac.uk (J.I.G.); Allison.Craig@icr.ac.uk (A.J.C.); 3 Department of Oncology, University of Oxford, Old Road Campus Research Building, Oxford OX3 7DQ, UK

**Keywords:** PET-CT, respiratory motion, SIRT, dosimetry, Yttrium 90

## Abstract

Respiratory motion degrades the quantification accuracy of PET imaging by blurring the radioactivity distribution. In the case of post-SIRT PET-CT verification imaging, respiratory motion can lead to inaccuracies in dosimetric measures. Using an anthropomorphic phantom filled with ^90^Y at a range of clinically relevant activities, together with a respiratory motion platform performing realistic motions (10–15 mm amplitude), we assessed the impact of respiratory motion on PET-derived post-SIRT dosimetry. Two PET scanners at two sites were included in the assessment. The phantom experiments showed that device-driven quiescent period respiratory motion correction improved the accuracy of the quantification with statistically significant increases in both the mean contrast recovery (+5%, *p* = 0.003) and the threshold activities corresponding to the dose to 80% of the volume of interest (+6%, *p* < 0.001). Although quiescent period gating also reduces the number of counts and hence increases the noise in the PET image, its use is encouraged where accurate quantification of the above metrics is desired.

## 1. Introduction

Selective internal radiation therapy (SIRT) is a treatment option for patients with tumours in the liver that cannot be surgically resected. SIRT is most commonly used to treat liver metastases from colorectal cancer and hepatocellular carcinoma, and involves injection of ^90^Y- or ^166^Ho-microspheres into the hepatic arterial vasculature [1,2]. Although SIRT has been in clinical use for over 15 years, the benefits of this treatment remain unclear. A review of SIRT in randomized controlled trials found a lack of evidence for improved survival or quality of life for colorectal cancer patients with metastatic disease in the liver [3]. Investigations into the efficacy of SIRT have demonstrated the existence of a strong dose–response relationship [4,5,6,7,8]. Hence, a personalised, optimised approach ensuring adequate tumour-absorbed dose may be crucial to increase the efficacy of the technique and to allow demonstrable and significant treatment benefits.

SIRT dose-response studies generally use ^90^Y PET imaging to provide estimates of tumour absorbed doses. Optimisation of the treatment, and dose verification, are hence reliant on accurate image-derived dosimetry [9]. Respiratory motion during the PET acquisition degrades the image quality. If left uncorrected, this leads to images with a blurring of the radioactivity distribution which will produce errors in dosimetry. In the case of SIRT, it is the motion of the liver that is of interest. This organ typically moves 10–26 mm cranio-caudally during normal respiration [10,11]. The purpose of the current investigation was to assess the effect of respiratory motion on tumour absorbed doses calculated from ^90^Y PET images following SIRT, and to evaluate the benefits of respiratory motion correction for this application.

The probability of positron production during ^90^Y decay is extremely low, making ^90^Y a challenging radionuclide to image using PET [12]. Despite relatively high activities in the scanner’s field of view (e.g., 0.5–3 GBq), the coincidence count-rate is low, hampering quantification. The quantitative accuracy of ^90^Y PET in the absence of motion has been evaluated by several groups [13,14,15,16] including a multi-centre phantom evaluation [17]. The consistent findings are that modern time-of-flight (TOF) PET scanners provide accurate quantification of radioactivity concentrations in phantoms, subject to the expected errors arising from a limited spatial resolution. Simulations performed by Ausland et al. [18] predicted that the errors in tumour dose quantification caused by respiratory motion could be substantial. The effects of image noise, respiratory motion, and motion compensations for ^90^Y SIRT have been considered by Siman et al. [19] who used short-duration images from the National Electrical Manufacturers Association (NEMA) International Electrotechnical Commission (IEC) body phantom, filled with ^18^F, to mimic noisy ^90^Y acquisitions. With no motion the quantification was found to be accurate, but large errors occurred when motion was applied (>50% for some dose measures), with these errors ameliorated by motion compensation (quiescent-period gating). Effects of respiratory motion have also been studied via simulation for pre-SIRT ^99m^Tc MAA SPECT [20]. Siman et al. [19] suggested that further studies, using anthropomorphic phantoms, with ^90^Y PET-CT studies, were needed to confirm the effectiveness of the motion compensation method. Our investigation addresses this by using clinically relevant activities of ^90^Y within an anthropomorphic phantom specifically designed for evaluation of SIRT dosimetry [21]. The phantom was filled and imaged several times to provide a range of activities and contrast ratios. Furthermore, we acquired data on two time-of-flight PET scanners from different vendors, allowing interpretation of the results in a wider context as opposed to being scanner-/vendor-specific. Respiratory gating signals were obtained using external devices, as data driven respiratory gating in clinical use for ^18^F and ^68^Ga based PET [22,23] has yet to be robustly implemented for ^90^Y PET [24].

## 2. Materials and Methods

### 2.1. Phantom Experiments

Experiments were performed on a Discovery D710 PET-CT scanner (GE; Milwaukee, WI, USA) and on a Biograph mCT (Siemens; Knoxville, TN, USA). The Abdo-Man^TM^ Phantom [21] was used as a test object throughout. This is a 3D-printed phantom of the abdomen, with a fillable liver compartment and fillable spherical inserts. The phantom was specifically designed to allow a SIRT-like radioactivity distribution to be generated within an object that mimics the lower-torso in terms of imaging characteristics (i.e., shape and density). Five inserts were placed within the liver region of the phantom, four of which were hollow fillable spheres of different diameter (10, 20, 30, 40 mm). The fifth insert was a 40 mm diameter sphere containing a 25 mm diameter solid inner sphere; this insert mimics the distribution of microspheres in the neovascular rim of a tumour that has a necrotic or poorly perfused core. Two of the spheres (the 40 mm with solid core, and the 20 mm diameter) were placed close to the superior end of the liver compartment. The 30 and 40 mm diameter spheres were close to the centre (in the superior-inferior direction) and the 10 mm diameter sphere was located near the inferior end of the compartment. All spheres were at least 10 mm from the boundaries of the liver compartment.

The phantom was filled with ^90^Y on four different occasions for each scanner. Four different total activities were used for each scanner, in the range 0.7–3.2 GBq. The spheres were filled with radioactivity concentrations that were greater than the surrounding liver compartment. Two sphere-to-liver concentration ratios were tested, 4:1 and 8:1. The total activity, activity concentrations and concentration ratios were accurately measured during phantom filling, through use of accurate scales and dose calibrators with calibration factors for ^90^Y that are traceable to the national standard. 

The phantom was placed on the QUASAR^TM^ respiratory motion platform (Modus QA; London, ON, Canada), which was set to be either stationary or to move according to a typical respiratory waveform. The platform translated the phantom axially to simulate the cranio-caudal motion of the liver during respiration. The maximum displacement from the central position was set to be ±10 or ±15 mm. Data were hence acquired with maximum displacements between inhalation and exhalation of 0, 20 and 30 mm. The respiratory motion system includes a platform that moves vertically in-time with the axial displacement of the phantom. This platform allows attachment of external devices used for monitoring respiratory motion, from which respiratory gating signals are obtained. For data acquired on the Discovery D710, the Real-time Position Management™ (RPM) Respiratory Gating system (Varian Medical Systems; Palo Alto, CA, USA) was used to track the position of a marker that was placed on the chest-wall platform. The system uses an infrared video camera for this purpose. For acquisitions on the Biograph mCT, gating signals were provided by a pressure belt on the same platform (Anzai Medical Corp.; Tokyo, Japan).

Following a helical CT scan, PET data were acquired for 15 min at a single bed position which included all spherical inserts. This acquisition protocol matches our local acquisition protocol for post-SIRT verification imaging [25]. Additional PET datasets were acquired with the phantom in motion, with motion amplitudes of 10 mm and 15 mm. For acquisitions with 10 mm motion amplitude the duration was increased, when possible, to 45 min and the data processed into 3 × 15 min images. This provided additional data to increase the power of the study. The process was repeated at four phantom activities on both PET-CT scanners. The total number of 15 min duration sets of raw data was 32. Data were reconstructed to provide attenuation and scatter corrected PET images. Different image reconstruction algorithms were tested for the Discovery 710, one based on suggestions from the QUEST study [17] as optimal for quantitative ^90^Y imaging, alongside a Bayesian penalized likelihood reconstruction. For the Discovery 710, the QUEST reconstruction was the manufacturer’s 3D OSEM reconstruction (VPFX) including resolution recovery (SharpIR) and TOF data, with two iterations of 24 subsets, without any z-axis filtration and using a 256 × 256 matrix size (giving voxels of 2.7 × 2.7 × 3.3 mm^3^). For the BPL (Q.Clear) reconstruction, a beta value of 1000 was used [25]. For the Biograph mCT, a local protocol was followed consisting of the manufacturer’s 3D OSEM reconstruction (1 iteration, 21 subsets) including resolution recovery and time of flight (without post filtering). For the cases where motion was present, images were reconstructed with and without quiescent period respiratory gating (QPG) as implemented by the manufacturers, retaining approximately 50% of the acquired data within the quiescent phase [26]. For the Discovery 710, the gating is phase-based (Q.Static™), while for the Biograph mCT it is amplitude-based (HD•Chest™).

### 2.2. Image Analysis

The images were analysed at a single site using commercial software (Hybrid Viewer v5.1, Hermes Medical Solutions AB, Stockholm, Sweden). The quantitative accuracy of the data was first verified for the stationary phantom acquisitions. For each image, 7 spherical volumes of interest (VOI), each 2 cm in diameter, were placed within the (background) liver compartment. These regions were placed at least 1 cm from the walls of the compartment and the inserts and were expected to be free of partial volume effects. We hence expected the mean radioactivity concentrations to closely match the known activity concentration, as determined from radionuclide dose calibrator measurements during preparation of the phantom.

The effects of respiratory motion on dosimetric measures were assessed by investigating the accuracy of activity quantification for the spherical inserts. Spherical VOIs were placed on each of the inserts, with the VOI manually centred on the given sphere as observed in the PET image. The VOI diameters matched the inner diameters of the spheres. In the case of the sphere with a solid core, two VOIs were analysed: one pertaining to the hot outer shell (a 40 mm spherical VOI but with a central 25-mm diameter sphere excluded), and one 25 mm spherical VOI centred on the cold core. These VOIs were repeated with manual alignment on each image from the study, i.e., for the phantom stationary or moving, with or without motion compensation via respiratory gating. From the hot spheres, an activity concentration volume histogram (ACVH) was extracted alongside the VOI mean activity concentration (AC_mean_). From these ACVHs, the dosimetric measure AC_80_ was extracted for summary analysis. AC_80_ is defined as the activity concentration threshold that incorporates 80% of voxels within the VOI. Although other thresholds can be used (e.g., AC_50_ as the median), we narrowed the current investigation to AC_80_ on the basis that we expect the mean or median dose to a tumour volume to have a weaker correlation with tumour response compared to AC_80_ [27], but higher thresholds such as AC_90_ to be more greatly affected by image noise. Results were converted to recovery percentages (i.e., the measured activity concentration as a percentage of the true activity concentration) to allow comparative analysis across the range of activity concentrations studied. The contrast recovery for the cold core was calculated as Q_C_ = (1 − C_core_/C_shell_) × 100%, where C_core_ is the VOI mean value of the cold core, and C_shell_ the VOI mean of the hot shell. Comparison of the results for the phantom in motion to those found with the phantom stationary, for the different sphere sizes, allowed the impact of respiratory motion and motion correction to be placed into perspective alongside the partial volume effect. Hence, the relative benefit of respiratory motion correction, in terms of impact on dosimetric measures, was assessed.

### 2.3. Statistical Analysis

The recovery coefficients from the four hot spherical inserts were used for a multiple linear regression analysis (IBM SPSS, v28) to determine which variables had a statistically significant impact on the measured recoveries. The impact of respiratory motion correction was further assessed by a paired, two-tailed *t*-test on the recovery coefficients from all inserts, comparing results from images with and without respiratory gating. The same test was applied to the AC_80_ values.

## 3. Results

The QUEST reconstruction for the Discovery 710 was found to be unsuitable for analyses at the voxel level and generation of an ACVH due to excessive noise. Hence, we focused our analysis on BPL images for this scanner.

**Figure 1:**
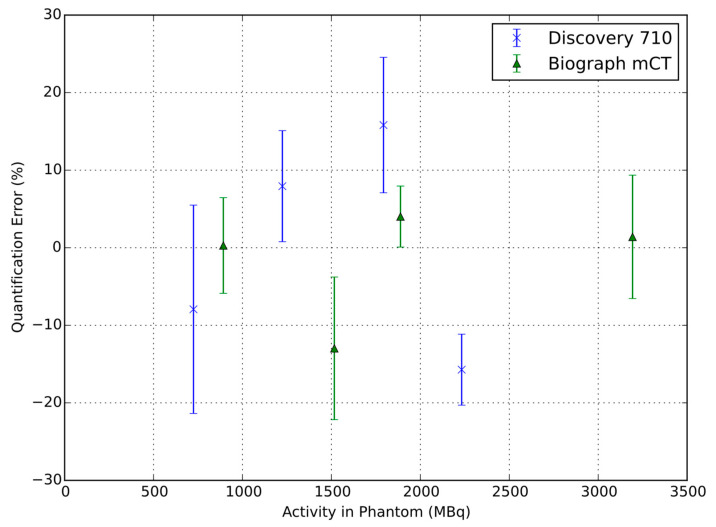
The quantification accuracy from the seven background regions in images from the Discovery 710 and Biograph mCT when the phantom was static. Error bars represent standard errors.

The quantitative accuracy of the images determined from the background region in the stationary phantom acquisitions, expressed as the percentage recovery coefficient, was 100.0 ± 7% and 98.2 ± 4% (mean ± standard error) for the GE Discovery 710 (using BPL reconstruction) and the Siemens Biograph mCT, respectively. This verifies that the phantom filling and scanner calibrations were accurate for ^90^Y. The quantification accuracy of the background region is presented in Figure 1.

A simple multi-linear model gave a reasonable fit to the recovery coefficients from the hot spheres, with the multiple correlation coefficient equalling 0.81 and the adjusted coefficient of determination being 0.65. The coefficients of the model were significantly different from zero for motion amplitude (*p* < 0.001) and motion correction (being on or off, *p* = 0.003). There was a dependence on the scanner (*p* < 0.001) and unsurprisingly on the sphere diameter (*p* < 0.001), the contrast (*p* < 0.001) and the phantom activity (*p* < 0.001). The model parameters confirmed an increased contrast recovery (+16% per cm) with increasing sphere diameter over the tested range of 1–4 cm. The application of 1–1.5 cm motion reduced the contrast (−11% per cm) which was partly compensated by motion correction being applied (+6.1% on average).

The paired-samples two-tailed *t*-test confirmed that the mean contrast recovery with quiescent period gating was significantly higher than without gating. For the Biograph mCT, the mean recovery coefficent, from all studies including motion and for all six VOI, was 55% without motion correction compared to 59% with respiratory gating (*p* = 0.007). For the Discovery 710, the corresponding values were 50% increasing to 55% with gating (*p* = 0.003). Similar findings were found when examining the AC_80_ values from the four hot spheres, where for the Biograph mCT the mean value of AC_80_ was 25% without motion correction compared to 29% with respiratory gating (*p* < 0.001). For the Discovery 710, the mean AC_80_ values were 23% and 29% for uncorrected motion and with respiratory gating respectively (*p* < 0.001).

**Figure 2:**
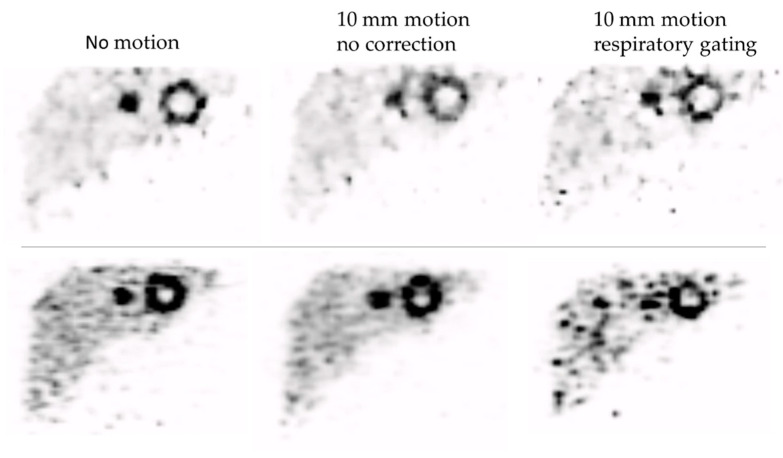
PET images (coronal slices) from the phantom with and without motion applied. The upper row of images is from the Discovery 710. The lower row is from the Biograph mCT. The grey scale maximum equals the true activity concentration in the hot spheres, with minimum at zero. The activity concentration ratio between the spheres and background was 8:1. For each scanner the images show a similar slice of the phantom while stationary, while in motion but without correction, and while in motion with quiescent period respiratory gating. The motion amplitude was 10 mm. The total activity in the phantom was 1.8 GBq for the Discovery and 1.9 GBq and Biograph. The slice contains the 40 mm insert with 25 mm cold core, as well as a 20 mm diameter sphere.

Example ^90^Y PET images are shown in Figure 2, where the improvement in contrast provided by gating is apparent at the expense of increased noise.

**Figure 3:**
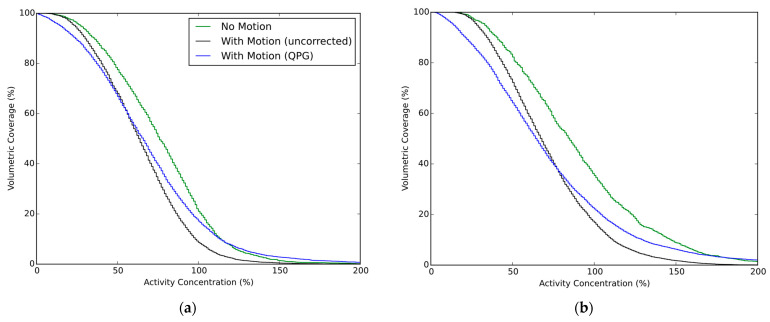
Activity concentration volume histograms for the 30 mm sphere. Each figure part shows the results for no motion, and when motion is present (10–15 mm amplitudes) either with or without quiescent period gating. Data are presented as the average ACVH for this sphere from the four phantom experiments on each scanner. (**a**) Discovery 710 (BPL reconstruction); (**b**) Biograph mCT.

**Figure 4:**
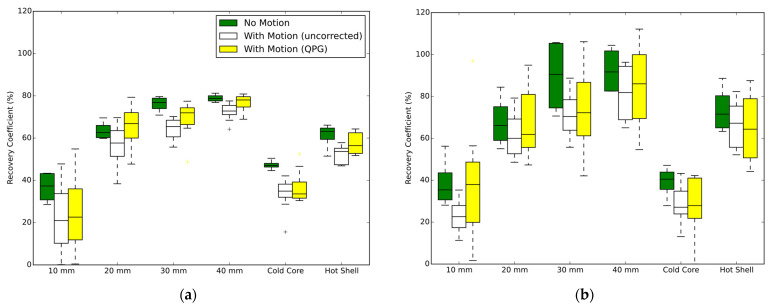
Contrast recoveries for different sphere diameters and types. Each figure part shows the results for no motion, and when motion is present (10–15 mm amplitudes) either with or without quiescent period gating. Combined datasets from four phantom experiments on each scanner. (**a**) Discovery 710 (BPL reconstruction); (**b**) Biograph mCT.

**Figure 5:**
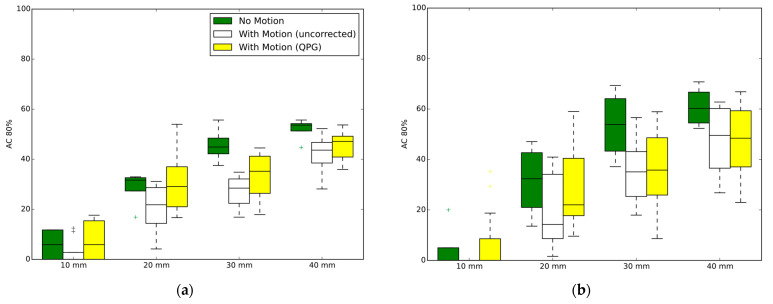
AC_80_ from different sphere diameters. Each figure part shows the results for no motion, and when motion is present (10–15 mm amplitudes) either with or without quiescent period gating. Combined datasets from four phantom experiments on each scanner. (**a**) Discovery 710 (BPL reconstruction); (**b**) Biograph mCT.

Averaged ACVHs are presented in Figure 3, for the case of the stationary and moving phantom with and without quiescent period gating. The recovery of the ^90^Y activity within the spherical inserts is presented in Figure 4 for which data are grouped by scanner and contrast ratio, with the presented data being the average of the tests performed at similar contrast ratios. The impact from the partial-volume effect for the different spheres is provided by the no-motion data, which is seen alongside the results for respiratory motion and respiratory motion with gating. The AC_80_ values extracted from the ACVHs are similarly presented in Figure 5.

## 4. Discussion

Using an anthropomorphic phantom filled with ^90^Y at clinically relevant activities placed on a respiratory motion platform, this investigation found that quiescent period respiratory gating leads to increased accuracy in measures of the activity concentration in both hot and cold features within a liver region. The study provided consistent results across a range of clinically relevant activities and at two contrast ratios, for two PET-CT scanners from different vendors. Our findings agree with expectations based on results from Siman et al. [19] who performed experiments using a less realistic phantom filled with a small amount of ^18^F on a single scanner. Although we found statistically significant increases in quantification accuracy, we note the data had high variance and the absolute gains from the application of quiescent period gating were moderate. The impact of the partial volume effect, seen by the difference in quantitative measures between smaller and larger spheres, was generally larger than the impact of applying the respiratory motion correction for the features analysed in this study (diameters ranging from 1 to 4 cm). This can be seen visually in Figure 4 and Figure 5 but was also evidenced by the coefficients of the multi-linear regression. A decrease in sphere diameter of 1 cm led to a larger decrease in contrast recovery (−16%) compared to respiratory motion of 1 cm amplitude (−11% and −5% for uncorrected data and for quiescent period gating, respectively).

In this study, and in agreement with Hou et al. [16], we noted that the QUEST reconstruction for the Discovery 710 was not suitable for a voxel-level analyses. We considered images with relatively high noise to be unsuitable for estimation of the distribution of activity concentrations within a region. This reconstruction should hence not be used when a dose-volume histogram analysis is to be performed. The quantitative accuracy of the QUEST reconstruction was, however, acceptable when assessed using the background region in the stationary phantom acquisitions, with a percentage recovery coefficient of 104 ± 8%. As the QUEST images from the Discovery 710 were too noisy to be subjected to ACVH analysis our work focused on the BPL images for this scanner. A limitation of our study is that we only studied images with β = 1000 as chosen based on previous investigations [25]. In the case of a voxel level analysis aiming for accurate estimation of the distribution of activity concentrations (for accurate DVH generation), high β values that provide smoother images may be optimal [16]. Furthermore, our study investigated only one method of respiratory motion correction (device-driven quiescent period gating) without optimisation of the retained fraction, which was set at 50%. This method of correction was chosen due to the relative simplicity and wide availability of the technique. The aim of our study was, however, not to optimise imaging parameters, but to demonstrate the extent to which respiratory motion degrades quantification accuracy, and the extent to which commonly used quiescent period gating can mitigate these effects. In this regard, our study shows that respiratory gating can be beneficial. Despite reducing the statistical quality of the images when discarding 50% of the coincidences, the application of gating was of net benefit with more accurate quantification of both VOI means and dosimetric measures such as AC_80_. However, we recognise the need for further investigations; for example, it would be useful from a practical point of view to define a minimum signal-to-noise ratio for the image (at the voxel level), below which the application of voxel-level dosimetric measures are not advised. Further development of methods for robust respiratory gating with retention of all (or most) counts in the case of ^90^Y PET is also needed. This is an ongoing area of research for ^18^F PET-CT imaging and the extension of methods, many of which use image registrations or other data-led techniques, to the case of low-count ^90^Y PET presents a variety of challenges. Without such techniques the justification for respiratory gating is tempered by the increase in image noise and the detrimental impact this has on voxel-level, DVH-type analyses. Increased smoothing or regularization of the image reconstruction (e.g., by increasing the β value in BPL) could compensate for the increased noise but at the expense of reducing spatial resolution. Although ^90^Y PET is challenging, we note that the current study did not utilise the most recent generation of long axial FOV, SiPM-based PET scanners. These offer significantly increased sensitivity through their extended axial coverage and reduced noise through improved time-of-flight capabilities, both of which make the newer scanners more suitable for gated ^90^Y PET. The image quality for gated studies is also expected to improve if higher activities are present in the scanner’s FOV. This study investigated the range of activities encountered at both sites (up to 3.2 GBq).

The use of respiratory gating is becoming more common, and it is noteworthy that in this patient population the additional radiation dose from a respiratory gated CT is likely justified given the poor prognosis of patients referred for SIRT. While data-driven respiratory gating methods for both ^90^Y PET and CT are yet to be robustly implemented in clinical practice, it may be appropriate to perform device-based gating of both the PET and the CT components of post-SIRT therapy PET-CT verification imaging. Accurate alignment of CT and PET images within the quiescent phase has been shown to be important for quantification using ^18^F and ^68^Ga PET-CT [28], and this is expected to apply equally to ^90^Y imaging.

As evidence mounts for a strong relationship between tumour dose and treatment response, the need for accurate dosimetry in SIRT also increases. While accurate dose estimation from pre-therapy imaging can be used to optimise treatment, post-therapy dosimetry can be used to verify the delivered dose and thus build the evidence base for the dose-response, allowing the treatment to be refined and to unravel the disease- and patient-related factors that may alter the dose thresholds for effective treatment.

## 5. Conclusions

Results from anthropomorphic phantom studies suggest that post-therapy SIRT PET-CT imaging is improved by quiescent period respiratory motion correction. Specifically, improved accuracy of tumour quantification and dosimetric measures are predicted.

## Data Availability

The data presented in this study are available on request from the corresponding author.

## References

[1] Salem R., Thurston K.G. (2006). Radioembolization with 90Yttrium Microspheres: A State-of-the-Art Brachytherapy Treatment for Primary and Secondary Liver Malignancies. Part 1: Technical and Methodologic Considerations. J. Vasc. Interv. Radiol. JVIR.

[2] Coldwell D., Sangro B., Salem R., Wasan H., Kennedy A. (2012). Radioembolization in the Treatment of Unresectable Liver Tumors: Experience across a Range of Primary Cancers. Am. J. Clin. Oncol..

[3] Townsend A.R., Chong L.C., Karapetis C., Price T.J. (2016). Selective Internal Radiation Therapy for Liver Metastases from Colorectal Cancer. Cancer Treat. Rev..

[4] van den Hoven A.F., Rosenbaum C.E.N.M., Elias S.G., de Jong H.W.A.M., Koopman M., Verkooijen H.M., Alavi A., van den Bosch M.A.A.J., Lam M.G.E.H. (2016). Insights into the Dose-Response Relationship of Radioembolization with Resin 90Y-Microspheres: A Prospective Cohort Study in Patients with Colorectal Cancer Liver Metastases. J. Nucl. Med..

[5] Fowler K.J., Maughan N.M., Laforest R., Saad N.E., Sharma A., Olsen J., Speirs C.K., Parikh P.J. (2016). PET/MRI of Hepatic 90Y Microsphere Deposition Determines Individual Tumor Response. Cardiovasc. Interv. Radiol..

[6] Kao Y.-H., Steinberg J.D., Tay Y.-S., Lim G.K., Yan J., Townsend D.W., Budgeon C.A., Boucek J.A., Francis R.J., Cheo T.S. (2013). Post-Radioembolization Yttrium-90 PET/CT—Part 2: Dose-Response and Tumor Predictive Dosimetry for Resin Microspheres. EJNMMI Res..

[7] Alsultan A.A., van Roekel C., Barentsz M.W., Smits M.L.J., Kunnen B., Koopman M., Braat A.J.A.T., Bruijnen R.C.G., de Keizer B., Lam M.G.E.H. (2021). Dose-Response and Dose-Toxicity Relationships for Glass 90Y Radioembolization in Patients with Liver Metastases from Colorectal Cancer. J. Nucl. Med..

[8] Garin E., Tselikas L., Guiu B., Chalaye J., Edeline J., de Baere T., Assenat E., Tacher V., Robert C., Terroir-Cassou-Mounat M. (2021). Personalised versus Standard Dosimetry Approach of Selective Internal Radiation Therapy in Patients with Locally Advanced Hepatocellular Carcinoma (DOSISPHERE-01): A Randomised, Multicentre, Open-Label Phase 2 Trial. Lancet Gastroenterol. Hepatol..

[9] Lea W.B., Tapp K.N., Tann M., Hutchins G.D., Fletcher J.W., Johnson M.S. (2014). Microsphere Localization and Dose Quantification Using Positron Emission Tomography/CT Following Hepatic Intraarterial Radioembolization with Yttrium-90 in Patients with Advanced Hepatocellular Carcinoma. J. Vasc. Interv. Radiol..

[10] Balter J.M., Ten Haken R.K., Lawrence T.S., Lam K.L., Robertson J.M. (1996). Uncertainties in CT-Based Radiation Therapy Treatment Planning Associated with Patient Breathing. Int. J. Radiat. Oncol. Biol. Phys..

[11] Clifford M.A., Banovac F., Levy E., Cleary K. (2002). Assessment of Hepatic Motion Secondary to Respiration for Computer Assisted Interventions. Comput. Aided Surg..

[12] D’Arienzo M. (2013). Emission of Β+ Particles Via Internal Pair Production in the 0+–0+ Transition of 90Zr: Historical Background and Current Applications in Nuclear Medicine Imaging. Atoms.

[13] van Elmpt W., Hamill J., Jones J., De Ruysscher D., Lambin P., Ollers M. (2011). Optimal Gating Compared to 3D and 4D PET Reconstruction for Characterization of Lung Tumours. Eur. J. Nucl. Med. Mol. Imaging.

[14] Carlier T., Willowson K.P., Fourkal E., Bailey D.L., Doss M., Conti M. (2015). (90)Y-PET Imaging: Exploring Limitations and Accuracy under Conditions of Low Counts and High Random Fraction. Med. Phys..

[15] Rowley L.M., Bradley K.M., Boardman P., Hallam A., McGowan D.R. (2017). Optimization of Image Reconstruction for 90Y Selective Internal Radiotherapy on a Lutetium Yttrium Orthosilicate PET/CT System Using a Bayesian Penalized Likelihood Reconstruction Algorithm. J. Nucl. Med..

[16] Hou X., Ma H., Esquinas P.L., Uribe C., Tolhurst S., Bénard F., Liu D., Rahmim A., Celler A. (2020). Impact of Image Reconstruction Method on Dose Distributions Derived from 90Y PET Images: Phantom and Liver Radioembolization Patient Studies. Phys. Med. Biol..

[17] Willowson K.P., Tapner M., QUEST Investigator Team, Bailey D.L. (2015). A Multicentre Comparison of Quantitative (90)Y PET/CT for Dosimetric Purposes after Radioembolization with Resin Microspheres: The QUEST Phantom Study. Eur. J. Nucl. Med. Mol. Imaging.

[18] Ausland L., Revheim M.-E., Skretting A., Stokke C. (2018). Respiratory Motion during 90Yttrium PET Contributes to Underestimation of Tumor Dose and Overestimation of Normal Liver Tissue Dose. Acta Radiol..

[19] Siman W., Mawlawi O.R., Mikell J.K., Mourtada F., Kappadath S.C. (2017). Effects of Image Noise, Respiratory Motion, and Motion Compensation on 3D Activity Quantification in Count-Limited PET Images. Phys. Med. Biol..

[20] Bastiaannet R., Viergever M.A., Jong H.W.A.M. (2017). de Impact of Respiratory Motion and Acquisition Settings on SPECT Liver Dosimetry for Radioembolization. Med. Phys..

[21] Gear J.I., Cummings C., Craig A.J., Divoli A., Long C.D.C., Tapner M., Flux G.D. (2016). Abdo-Man: A 3D-Printed Anthropomorphic Phantom for Validating Quantitative SIRT. EJNMMI Phys..

[22] Kesner A.L., Chung J.H., Lind K.E., Kwak J.J., Lynch D., Burckhardt D., Koo P.J. (2016). Validation of Software Gating: A Practical Technology for Respiratory Motion Correction in PET. Radiology.

[23] Walker M.D., Morgan A.J., Bradley K.M., McGowan D.R. (2020). Data-Driven Respiratory Gating Outperforms Device-Based Gating for Clinical 18F-FDG PET/CT. J. Nucl. Med..

[24] Chiesa C., Sjogreen-Gleisner K., Walrand S., Strigari L., Flux G., Gear J., Stokke C., Gabina P.M., Bernhardt P., Konijnenberg M. (2021). EANM Dosimetry Committee Series on Standard Operational Procedures: A Unified Methodology for 99mTc-MAA Pre- and 90Y Peri-Therapy Dosimetry in Liver Radioembolization with 90Y Microspheres. EJNMMI Phys..

[25] Scott N.P., McGowan D.R. (2019). Optimising Quantitative 90Y PET Imaging: An Investigation into the Effects of Scan Length and Bayesian Penalised Likelihood Reconstruction. EJNMMI Res..

[26] Liu C., Alessio A., Pierce L., Thielemans K., Wollenweber S., Ganin A., Kinahan P. (2010). Quiescent Period Respiratory Gating for PET/CT. Med. Phys..

[27] Dewaraja Y.K., Schipper M.J., Roberson P.L., Wilderman S.J., Amro H., Regan D.D., Koral K.F., Kaminski M.S., Avram A.M. (2010). 131I-Tositumomab Radioimmunotherapy: Initial Tumor Dose-Response Results Using 3-Dimensional Dosimetry Including Radiobiologic Modeling. J. Nucl. Med..

[28] Thomas M.A., Pan T. (2021). Data-Driven Gated PET/CT: Implications for Lesion Segmentation and Quantitation. EJNMMI Phys..

